# Felty syndrome with liver cirrhosis: A case report

**DOI:** 10.1097/MD.0000000000042116

**Published:** 2025-04-25

**Authors:** Chengyi Wan, Mei Wu, Limei Jiang, Chaofeng Zhang, Guolin Gan, Xuehua Sun

**Affiliations:** a Department of Liver Diseases, Shuguang Hospital Affiliated to Shanghai University of Traditional Chinese Medicine, Shanghai, China; b Department of Traditional Chinese Medicine, Shanghai Putuo District People’s Hospital, Shanghai, China; c Department of Traditional Chinese Medicine, Shanghai Pudong New Area Shanggang Community Health Service Center, Shanghai, China; d Department of Traditional Chinese Medicine, Naval Medical Center, Shanghai, China.

**Keywords:** case report, felty syndrome, literature review, liver cirrhosis, rheumatoid arthritis

## Abstract

**Rationale:**

Felty syndrome (FS) is a rare type of rheumatoid arthritis, and its combined occurrence with liver cirrhosis was rarely reported. FS was easily misdiagnosed as autoimmune cirrhosis or myelodysplastic syndrome, which led to improper medication and serious consequences.

**Patient concerns:**

A 72-year-old male patient was admitted to Shuguang Hospital Affiliated with the Shanghai University of Traditional Chinese Medicine due to recurrent fatigue associated with a cough. Imaging suggested liver cirrhosis and splenomegaly, according to imaging diagnosis, laboratory tests, ultrasound, magnetic resonance, and history of rheumatoid arthritis, we considered the diagnosis of liver cirrhosis due to FS. Immunomodulatory and anti-liver fibrosis therapy was carried out, combined with Chinese patent medicines, and the patient’s condition was stable in the future years.

**Diagnoses:**

FS, liver cirrhosis, and chronic renal insufficiency.

**Interventions:**

Routine immunomodulation, liver protection, and anti-liver fibrosis treatment, moreover Traditional Chinese Medicine was used to protect the liver and promote bile excretion, and regulate immunity.

**Outcomes:**

The patient’s symptoms improved and his subsequent condition stabilized.

**Lessons:**

Physicians should have a better understanding of FS and know that it can lead to liver cirrhosis, to avoid misdiagnosis and poor response to glucocorticoids, delay the disease, and increase the burden on patients.

## 1. Introduction

Manifestations of Felty syndrome (FS) were firstly reported in 1924, and later, Dr Hanrahan termed the triad of rheumatoid arthritis (RA), neutropenia, and splenomegaly as FS in 1932.^[1]^ FS is a clinically rare form of RA. Notably, its incidence in whites is higher than that in blacks, and the ratio of males to females is approximately 1:3.^[2]^ FS is accompanied by hepatic nodular regenerative proliferation, which is rarely observed in lupus erythematosus and other connective tissue diseases.^[3]^ Histopathological abnormalities and abnormal liver function has been confirmed in 60% of the patients with FS.^[1]^ However, FS accompanied by liver cirrhosis was rarely reported. In this study, we reported a case with FS accompanied with liver cirrhosis.

## 2. Case presentation

**Figure 1.**
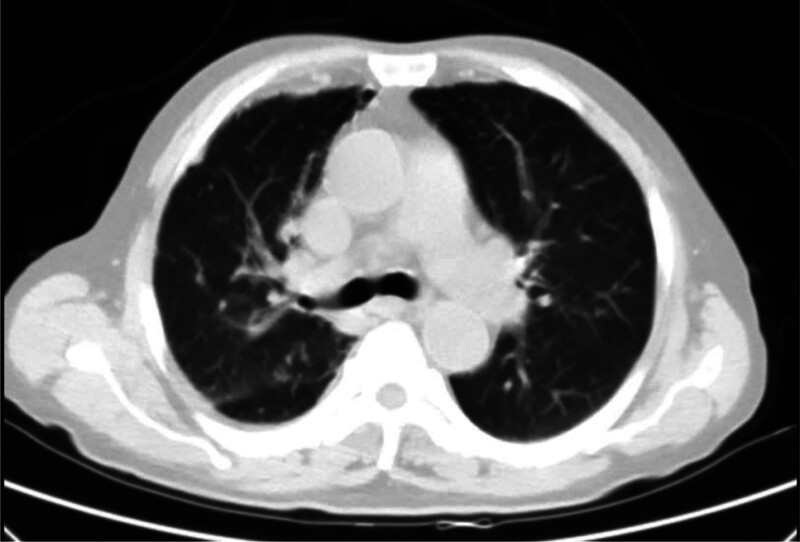
Chest CT showed interstitial changes in both lungs with chronic inflammation. CT = computed tomography.

A 72-year-old male patient visited Shuguang Hospital Affiliated to Shanghai University of Traditional Chinese Medicine in February 2019 due to a fever (37.6°C), cough, and sputum for 3 days, after the patient had caught a cold. Upon admission, he had a cough, expectoration, white sputum decrease, and occasional fatigue. Based on medical history, the patient developed joint pain and was limited mobility in 2001. He was diagnosed with RA and treated with leflunomide and voltaren in Renji Hospital, Shanghai. The joint pain was relieved, and the movement was improved. After discharge, he continued regularly taking leflunomide. He has stopped taking them for 2 years till now. In July 2008, joint deformity, splenomegaly, and leukopenia were observed whereas not properly treated. In March 2012, the computed tomography (CT) showed that the kidney cortex was not smooth; sequelae of inflammation were present; the density of the liver was uniform, but splenomegaly was manifested. In June 2016, physical examination at Renji Hospital suggested that the right kidney was small and the images of both kidneys showed injuries. No obvious abnormality was observed in the bilateral ureters. In March 2017, renal insufficiency was diagnosed, with the following laboratory parameters: urea nitrogen 8.3 mmol/L↑; creatinine 118.8 µmol/L↑; uric acid 761.00 µmol/L↑. The treatment procedure was unknown. In August 2018, the patient’s Chest CT scan revealed liver cirrhosis and splenomegaly. In 2019, his fingers and toe joints are deformed, without obvious swelling and pain, and did not take other drugs recently. Denied the history of hypertension, chronic cough and asthma, coronary heart disease, and other chronic diseases in internal medicine. Denied the history of infectious diseases, such as tuberculosis and typhoid fever. His admission diagnoses were acute upper respiratory tract infection, liver cirrhosis (unknown causes), splenomegaly, RA, and chronic renal insufficiency. Physical examination shown body temperature: 37.5℃, pulse: 78 times/ min, breath: 20 times/min, blood pressure: 130/70 mm Hg. Tonsil body II degree was enlarged. Two lungs were cleared by percussion, and the breathing sound was still clear. Liver subcostal ribs was not touched, subsplenic ribs can be touched 1 to 2 fingers, mobile turbidity sound (–). The metacarpophalangeal (MCP) joints, bilateral wrist joints, bilateral elbow joints, and bipedal metatarsophalangeal joints are deformed, with adverse movement, no pain and tenderness in the limb joints. The muscle strength and muscle tone of the limbs are normal. Laboratory tests was shown in Table 1. In addition, ANA, ENA, liver disease autoantibodies, IgG4 (−); thyroid function (−); hepatitis virus markers, HIV, RPR (−); ceruloplasmin (−); tumor (−); stool routine (−). Chest CT showed interstitial changes in both lungs with chronic inflammation (Fig. 1). Small solid nodules in the upper and middle lobe of the right lung were observed, with chronically enlarged inflammatory nodules. Coronary artery calcification, liver cirrhosis, and splenomegaly were also observed.

**Table 1:** Collection of patient symptoms and examinations from 2002 to 2021.

Time		Symptoms		Ultrasound		Fibroscan		MRI		RBC (10^12^/L)		WBC (10^9^/L)		Neu (10^9^/L)	
September 2001		Joint pain and limited mobility		/		/		/		–		–		–	
July 2008		Joint deformity and limited mobility		Normal liver; splenomegaly		/		/		–		↓#		↓#	
March 2012		Joint deformity and limited mobility; abdominal distension and fatigue		Normal liver; splenomegaly		/		/		–		↓#		↓#	
June 2016		Abdominal distension and fatigue		Normal liver; splenomegaly; double kidneys injury		/		/		4.11 ↓		4.86 ↓		3.60 ↓	
August 2018		Joint deformity and limited mobility; abdominal distension and fatigue		Normal liver; splenomegaly; double kidneys injury		/		/		3.64 ↓		4.60 ↓		4.90 ↓	
February 2019		Fever and cough; joint deformity and limited mobility; abdominal distension and fatigue		Liver cirrhosis; splenomegaly		/		/		2.99 ↓		6.48		5.80	
March 2019		Joint deformity and limited mobility; abdominal distension and fatigue		Liver cirrhosis; rough gallbladder wall; splenomegaly (71 × 166 mm); SPV: 14 mm; splenic veins were widened; both kidneys were small		TE: 27.4 kPa; CAP: 221 dB/m		Liver cirrhosis; Gallbladder slightly larger; kidney atrophy and small cysts		3.06 ↓		2.88 ↓		1.66 ↓	
June 2021		Joint deformity and limited mobility; abdominal distension and fatigue improved		Liver cirrhosis; rough gallbladder wall; splenomegaly (61 × 132 mm); SPV: 13mm; splenic veins were widened; both kidneys were small		TE:16.5kPa; CAP: 202 dB/m		–		2.03 ↓		3.45 ↓		1.16 ↓	

PLT (10^9^/L)	Hb (g/L)	CRP (mg/L))	ALT (U/L)	AST (U/L)	GGT (U/L)	ALP (U/L)	TBil (µmol/L)	Alb (g/L)	Scr (µmol/L)	UA (µmol/L)	GFR (mL/min*1.73 m^2^)	RF (IU/mL)	IgG (g/L)	ENA	Anti-cyclic citrullinated peptide
–	–	–	–	–	–	–	–	–	–	–	–	–	–	–	/
↓#	/	/	/	/	/	/	/	/	/	/	/	/	/	/	/
↓#	/	/	/	/	/	/	/	/	/	/	/	/	/	/	/
77 ↓	124 ↓	/	/	/	/	/	/	/	160 ↑	739 ↑	35.7↓	/	/	/	+
59 ↓	114 ↓	0.94 ↑	51	77 ↑	143 ↑	271 ↑	28.5	32.0↓	149 ↑	775 ↑	36.9 ↓	394 ↑	20 ↑	–	–
63 ↓	94 ↓	52.41 ↑	31 U/L	51 ↑	66 ↑	180 ↑	23.4	27.6↓	220↑	640 ↑	23.2 ↓	333 ↑	20 ↑	–	–
74 ↓	95 ↓	10.11 ↑	27 U/L	12	57	212 ↑	14	26.8 ↓	166 ↑	590 ↑	31.3 ↓	305 ↑	22.9 ↑	–	+
92 ↓	108 ↓	2.61	14	19	22	138	18.6	30.9↓	161 ↑	420 ↑	31.3 ↓	202 ↑	17.3	–	/

+ represents positive, – represents negative, / represents the patient has not have this examination, # represents the patient did not provide the specific value.

Alb = albumin, ALP = alkaline phosphatase, ALT = alanine aminotransferase, AST = aminotransferase, CAP = controlled attenuation parameter, CRP = C-reactive protein, ENA = perinuclear type, GFR = glomerular filtration rate, GGT = gamma-glutamyl transpeptidase, Hb = hemoglobin, MRI = magnetic resonance imaging, Neu = neutrophile granulocyte, PLT = platelets, RA = rheumatoid arthritis, RBC = red blood cell, RF = rheumatoid factor, Scr = serum creatinine, SPV = splenic vein diameter, TBiL = total bilirubin, TE = transient elastography, UA = uric acid, WBC = white blood cell.

**Figure 2.**
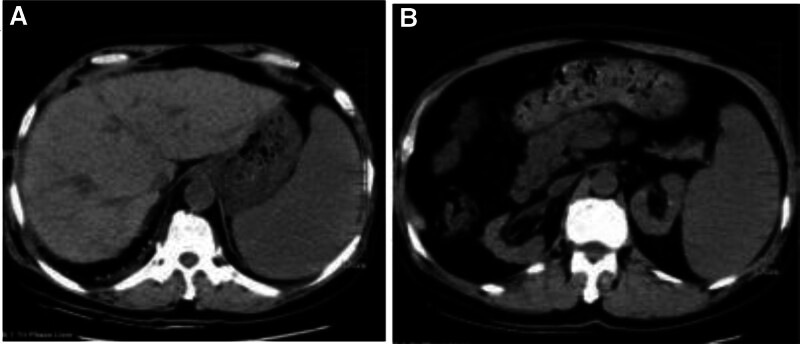
(A and B). Upper abdominal CT showed liver cirrhosis, splenomegaly, and portal hypertension. CT = computed tomography.

**Figure 3.**
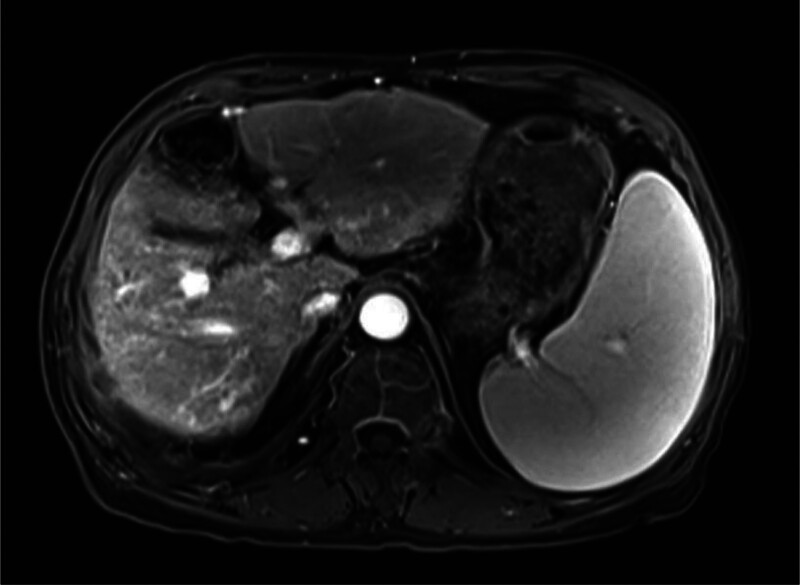
Upper abdominal MRI showed the gallbladder was slightly larger, which might have been caused by liver cirrhosis; kidney atrophy and small cysts were also observed. MRI = magnetic resonance imaging.

**Figure 4.**
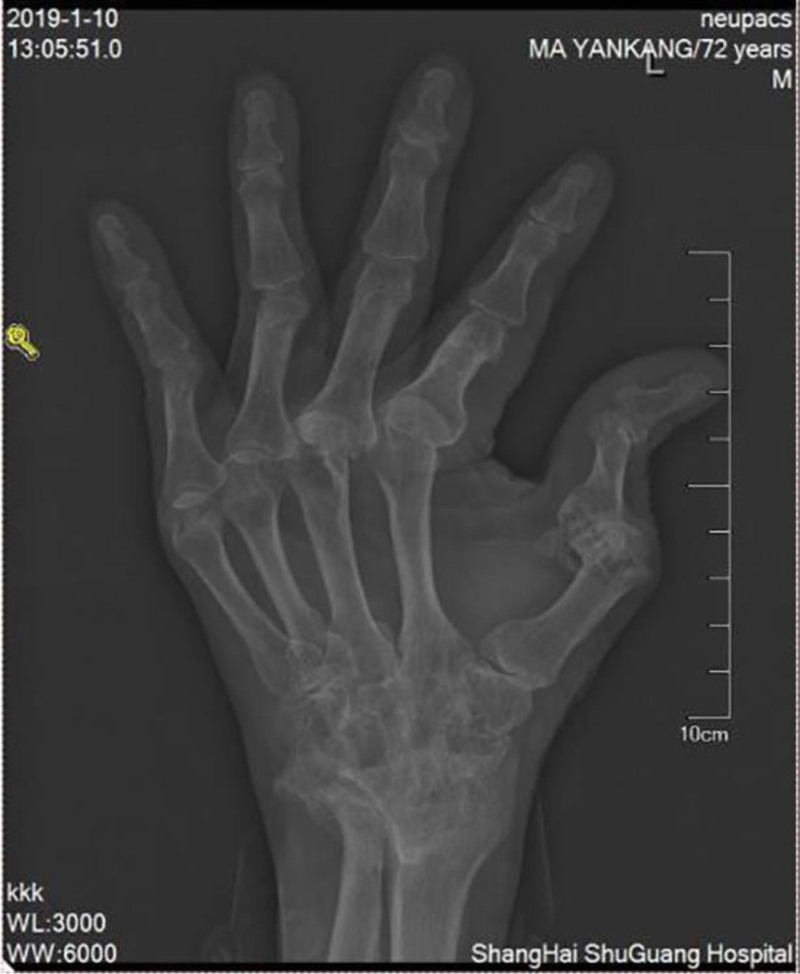
Plain radiographs of limb joints showed MCP2 bone erosion in the left hand.

**Figure 5.**
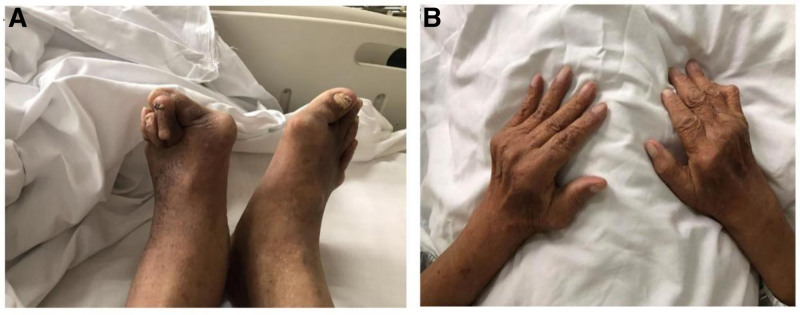
(A) Photos of bipedal joints; (B) photo of the joints of both hands. Joints deformation were very obvious.

**Figure 6.**
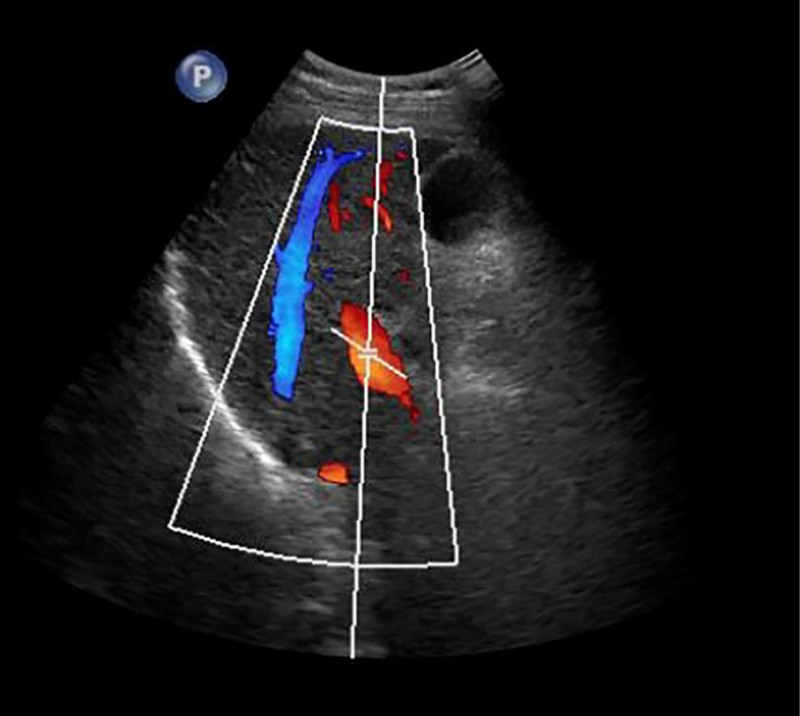
Abdominal ultrasound observed no obvious fluid accumulation in the abdominal cavity, the portal vein presented smooth blood circulation in the portal vein system, and the splenic veins widened.

**Figure 7.**
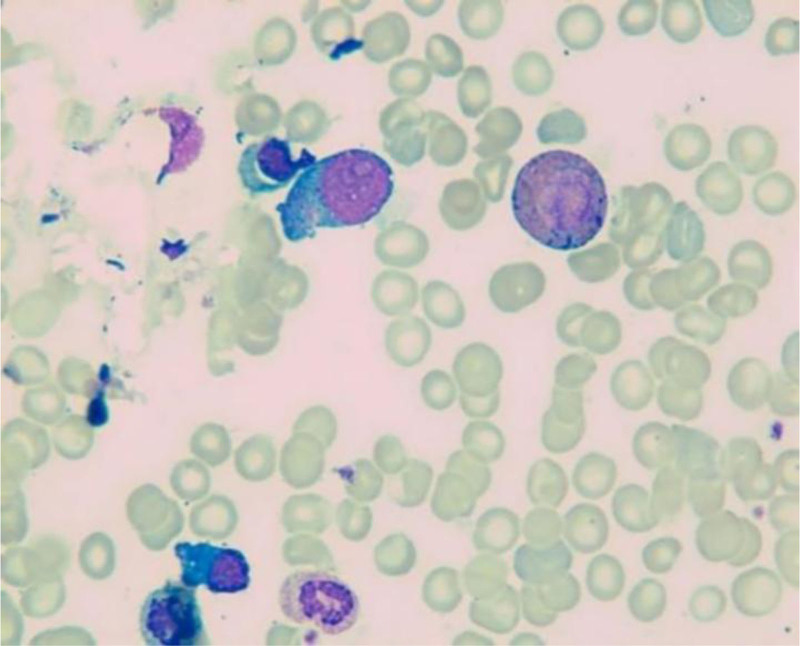
Bone marrow smear indicated active nucleated cell proliferation, active granular cells, red cell and macrophage proliferation, and normal morphological characteristics.

**Figure 8.**
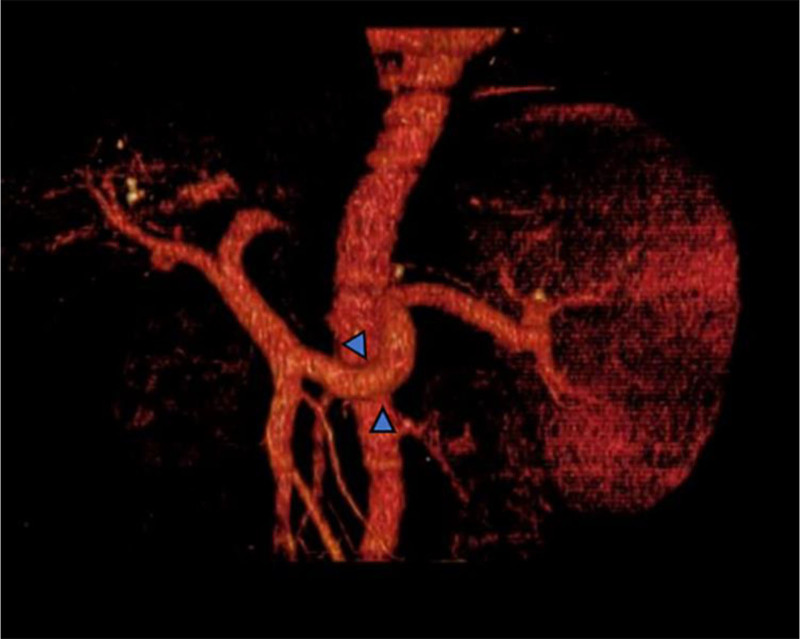
A portal vein in CTA showed portal vein hypertension and splenic portal vein varicose. CTA = computed tomography angiography.

He was treated with glutathione, polyene phospholipid choline, hepatocyte growth factor, pioneer metarol, mucosultan, asimer, ricotine, and Shuganning injections. He was discharged after clinical symptoms were alleviated. In March 2019, the patient was rehospitalized due to repeated fatigue for more than 1 month. Liver and kidney function test results are presented in Table 1. A plain CT scan of the upper abdomen showed liver cirrhosis, splenomegaly, and portal hypertension (Fig. 2A and B). The gallbladder was slightly larger, which might have been caused by liver cirrhosis; kidney atrophy and small cysts were also observed (Fig. 3). Fibroscan findings showed transient elastography: 27.4 kPa, controlled attenuation parameter: 221 dB/m. Plain radiographs in the extremities and joints revealed MCP degeneration in both hands, MCP2-4 bone erosion in the right hand, MCP2 bone erosion in the left hand, MCP2 in the right hand with severe synovial hyperplasia and mild synovitis, MCP3 with severe synovial hyperplasia and moderate synovitis, MCP4 moderate synovial hyperplasia and mild synovitis, MCP2 mild synovial hyperplasia and mild synovitis in both hands and PIP degeneration in both hands. Degeneration in both wrist joints, bone erosion, moderate synovial hyperplasia in the left wrist joint, and mild synovitis with effusion were observed. The right wrist joint is accompanied with mild synovial hyperplasia and mild synovitis with effusion. Degeneration and bone erosion were present in the elbow joints. The right elbow joint is accompanied with moderate synovial hyperplasia with mild synovitis and less effusion. The left elbow joint presented less effusion but was with severe synovial hyperplasia with mild synovitis. Joints deformation were very obvious (Figs. 4, 5A and B). Abdominal ultrasound showed diffuse liver parenchymal lesions (liver cirrhosis), rough gallbladder wall, splenomegaly (71 × 166 mm), and widened splenic vein diameter (SPV: 14 mm). Both kidneys were small and with chronic kidney disease-like manifestations. We also observed a left renal cyst with no obvious fluid accumulation in the abdominal cavity. The Portal vein presented smooth blood circulation in the portal vein system. The splenic veins were widened (Fig. 6). Bone marrow examination indicated active nucleated cell proliferation, active granular cells, red cell and macrophage proliferation, and normal morphological characteristics (Fig. 7). Electronic gastroscopy showed congestive and exudative gastritis, hyperplasia of Brucella glands in the duodenum, and protruding changes in the posterior wall of the gastric body. Computed tomography angiography showed portal vein hypertension and splenic portal vein varicose, liver cirrhosis, splenomegaly, small liver cyst, left kidney cyst, small low-density nodules, and small vascular tumors in the spleen (Fig. 8).

To summary, the patient initially presented with joint symptoms, splenomegaly, and leukopenia in 2008. In 2012, a CT scan revealed splenomegaly with no abnormalities detected in the liver. In 2018, the patient exhibited liver cirrhosis and severe portal hypertension. The results of the liver and spleen function in past years are summarized in Table 1. According to the literature this patient meets the diagnostic criteria for FS with RA; splenomegaly; platelets (PLT): 63 × 10^9^/L; no other reasons explaining splenomegaly or granulocytopenia.^[4]^ We inferred that this clinical progression resulted from congestive splenomegaly caused by FS, which led to portal venous flow obstruction and subsequent cirrhosis. Notably, this pathological mechanism is diametrically opposed to that of cirrhosis originating from primary liver disease. Based on the history and symptoms, FS caused portal hypertension, which leads to the appearance of cirrhosis. Therefore, based on the medical history and the symptoms, he was diagnosed with RA, FS, liver cirrhosis, and chronic renal insufficiency [chronic kidney disease (CKD) phase III–IV]. He was treated with Metola 2 mg (bid), which was then reduced to 2 mg (qd) for maintenance. Febuxostat 10 mg (qd); Pavlin tablets; Huangkui capsules, Jinshuibao; Atomolan tablets; ursodeoxy bile acid; Likejun and Traditional Chinese medicine, like Fuzheng Husyu formula. In May 2020, a sinus was formed due to the rupture of the left buttock nodule. The patient healed after being filled with red oil ointment gauze and drained. The latest follow-up examination showed that the disease has been stable. The patient agrees with the current treatment. In June 2021, a reexamination was performed in Table 1. Fibroscan: transient elastography:16.5 kPpa, controlled attenuation parameter: 202 dB/m, spleen size (61 × 132 mm), widened splenic vein diameter (SPV: 13 mm).

## 3. Discussion

Felty syndrome could be asymptomatic, most of which presents RA-related joint disease and other manifestations, such as weakness, weight loss, liver cirrhosis, small vasculitis, and recurrent infections. Among them, severe local or systemic infection may be the first clue to the diagnosis of FS and the main cause of death. The most common infections include those of the skin, oral cavity, upper respiratory tract, lower respiratory tract, and the urinary tract. At a neutrophil count lower than 500/μL, patients are more susceptible to infection, leading to a high incidence of bacterial infection.^[1]^ Initially, patient in the present case was diagnosed with unexplained liver cirrhosis due to upper respiratory infection. Later, the history of RA was traced back for more than 10 years. In the early onset stage, he had symptoms such as neutropenia, splenomegaly, and renal insufficiency. He had taken kinds of drugs and was autoimmune antibody-positive, and thus his condition needed to be differentiated from drug-induced liver cirrhosis, autoimmune cirrhosis, and blood system diseases.

In this case, laboratory examination, magnetic resonance imaging, bone puncture, patient’s the Roussel Uclaf Causality Assessment Method score, and autoimmune hepatitis score did not meet the criteria for the diagnosis of drug-induced liver and autoimmune liver disease. The bone puncture indicated that the bone marrow hyperplasia was normal, and hematological diseases were ruled out. The portal vein computed tomography angiography, B-ultrasound, and gastroscopy showed that the patient had splenomegaly, splenic varices, and portal hypertension. There was no obvious abnormality in the liver function, which was different from the clinical manifestations of liver cirrhosis and portal hypertension caused by liver disease. The patient initially presented with extrahepatic manifestations including arthritis, splenomegaly, and granulocytopenia, which was followed by the development of portal hypertension, with liver cirrhosis ultimately manifesting as the terminal complication of the disease. This progression suggests that FS induced congestive splenomegaly, which subsequently caused portal venous flow obstruction and ultimately led to the development of liver cirrhosis. Therefore, combined with medical history data, we considered that the FS was the main disease cause, 3-line decline (white blood cell, red blood cell, and hemoglobin), splenomegaly, splenic varices, and portal hypertension, and then splenic liver cirrhosis. Finally, the patient was treated with Total Glucosides of Paeony capsules regulated immunity and Fuzhenghuayu capsule stasis against liver fibrosis. Due to the long course of the disease, multiple complications, and causal interactions, the diagnosis of FS is complicated.

A small number of patients with this disease could be relieved naturally and were asymptomatic for a few years, whereas the possibility of self-healing is extremely low.^[5]^ Meanwhile, the risk of severe infection in patients with FS is increased, the overall mortality rate is high, and the prognosis is poor. Currently, the following main strategies for FS treatment are applied, which are basically the same as those used for RA: nonsteroidal anti-inflammatory drugs and antirheumatic drugs (such as methotrexate, leflunomide, sulfasalazine, and hydroxychloroquine); Immune and biological agents (such as cyclophosphamide, tumor necrosis factor-α blocker, and rituximab); corticosteroids; granulocyte colony-stimulating factor; splenectomy; other (traditional Chinese medicine). Methotrexate is widely used as the initial treatment for patients with RA and neutropenia and is considered to be a conventional treatment. During RA therapy, local or systemic infections and immunosuppression tend to make patients susceptible to the development of potentially dangerous complications, which limits the use of immunosuppressive agents. Different effectiveness of cyclophosphamide and biological agents was observed, whereas they can induce neutropenia and increase the risk of infection. Thus, their use was limited. Corticosteroid therapy is not considered a reliable treatment option for neutropenia in RA. However, clinical reports still support its effectiveness.^[6]^ Nevertheless, the prevention of infection aggravation should be carefully considered. Although there are case reports that the granulocyte colony-stimulating factor was effective for severe neutropenia treatment, currently, randomized clinical trials for the treatment of neutropenia and its infectious complications are lacking, and its reliability is, hence, questionable.^[7]^ Splenectomy is the last treatment for patients with severe neutropenia and repeated FS infection, but its effectiveness is not long-lasting. Traditional Chinese medicine could be considered as a supplemental strategy when the above-mentioned single regimen treatment had failed. Because there is no specific drug for this kind of patients, we considered comprehensive treatment. In addition to the medications we continue to use to control RA, we use Traditional Chinese medicine. Fuzheng Huayu were useful to treat liver cirrhosis which developed by Shuguang Hospital affiliated with Shanghai University of Traditional Chinese Medicine. It consists of Radix Salvia miltiorrhiza (Danshen), Persicae semen (Taoren), Cordyceps (Dongchongxiacao), Gynostemma pentaphylla (Jiaogulan), Schisandrae chinensis fructus (Wuweizi) and Pini pollen (Songhuafen). It has conducted phase II clinical trials under FDA approval. It can improve liver fibrosis, alleviate patient symptoms, and demonstrate favorable clinical efficacy.^[8]^ Basic research showed that it has anti-fibrotic effect by inhibiting hepatic stellate cell activation, reducing inflammation, protecting hepatocytes, inhibiting hepatic sinusoidal capillary formation and angiogenesis, promoting extracellular matrix degradation and promoting liver regeneration.^[9,10]^ In this case, the patient benefited from comprehensive treatment, and his liver stiffness changed from 27.4 to 16.5 kPa, and his condition remained stable. This therapy reduces the occurrence of fatigue and ascites effusion.

## 4. Conclusion

Felty syndrome is a rare and specific type of RA. However, in addition to the joint disease (Foreign researchers reported that no initial extra-articular manifestations might be present in some cases). The clinical manifestations are atypical and with diversified characteristics, which are often affected by other hidden factors. These issues could hinder the diagnosis of FS, preventing the timely clinical diagnosis of FS. Therefore, FS should be considered, if the patient has had a history of RA, neutropenia, splenomegaly, or even unexplained liver cirrhosis (liver involvement caused by FS is not common.), and repeated local or systemic (past or current) infections, especially when other pathological causes for neutropenia with RA have been excluded. In addition, clear diagnostic indicators and therapeutic drugs need to be further investigated.

## Acknowledgments

The authors thank all the staff and patient for their valuable contribution to the study.

## Author contributions

**Conceptualization:** Xuehua Sun.

**Data curation:** Chengyi Wan, Limei Jiang, Chaofeng Zhang, Guolin Gan.

**Methodology:** Mei Wu.

**Writing – original draft:** Chengyi Wan.

**Writing – review & editing:** Mei Wu.
